# Queering reproductive access: reproductive justice in assisted reproductive technologies

**DOI:** 10.1186/s12978-021-01214-8

**Published:** 2021-08-02

**Authors:** Michelle W. Tam

**Affiliations:** Dalla Lana School of Public Health, University of Toronto, 155 College St Room 500, Toronto, ON M5T 3M7 Canada

## Abstract

**Background:**

Advancements in assisted reproductive technologies (ART) and policy development have enabled more people to have biologically related children in Canada. However, as ART continues to focus on infertility and low fertility of heterosexual couples, ART access and research has been uneven towards meeting the reproductive needs of lesbian, gay, bisexual, transgender, queer, two-spirit, intersex, and asexual (LGBTQ2SIA +) people. Furthermore, experiences of reproduction are impacted by intersectional lived realities of race, gender, sexuality, and class. This commentary utilizes a reproductive justice (RJ) framework to consider reproductive access for LGBTQ2SIA + Black, Indigenous, and people of colour (BIPOC), while simultaneously engaging through a critical lens RJ has on ART. An RJ framework considers the constitutive elements of reproductive capacity and decision making that are not often at the forefront of reproductive health discussions. Additionally, this commentary discusses reproductive rights violations and reproductive violence such as coerced and forced sterilizations that have and are currently occurring in Canada. This article considers systems of access and structures of regulation that seek to control the reproductive capacities of marginalized communities, while empowering accessibility and upholding white supremacy and heteronormativity. In thinking through research and access in ART, who are ART users and whose reproduction is centered in research and access in Canada?

**Conclusion:**

A reproductive justice framework is urgently needed to address inequities of sexual and reproductive health access in Canada.

## Background

Evidence indicates a significant increase in the use of assisted reproductive technologies (ART) in Canada over the past two decades [1, 2]. These technological innovations have helped heterosexual couples [2], lesbian, gay, bisexual, transgender, queer, and two-spirit (LGBTQ2S+) parents, and single parents to have biologically related children and in turn, expand ideologies around nuclear family building. However, despite these social and biomedical innovations in the field of reproductive health, access to ART continues to be uneven across intersecting social categories of race, gender, sexuality, and class [3–6].

To address complex issues in ART access and research, it is necessary to utilize a framework that centers complexity and lived realities constituting reproductive capacity and decision making outside of predominant white heteronormative standards. Adopting a reproductive justice framework offers a more nuanced approach to address sexual and reproductive health inequities among Black, Indigenous and people of colour (BIPOC) with intersectional lived experiences in Canada. Further, in order to meaningfully engage and advocate for the implementation and use of a reproductive justice framework within sexual and reproductive health research (SRHR) and ART, historical and present injustices of reproductive violence must be acknowledged and addressed.

## Reproductive justice framework

Rooted in Black feminist thought, critical race theory, and critical feminist theory [7–9], reproductive justice (RJ) is an interdisciplinary framework that combines reproductive rights and social justice [10]. RJ recognizes reproductive oppression as inherently interconnected with multiple intersections of oppression including racism, classism, sexism, homophobia, health status, and access to healthcare [10, 11]. Beyond an analytical framework, RJ is a movement aimed at challenging white supremacist structures and systems that control populations through regulation of bodies, sexuality, labour, and reproduction [10]. The movement is concerned with the day-to-day conditions constituting a person’s reproductive decisions including access to resources and healthcare, familial elements, cultural values, educational opportunities, and other familial and community needs [10].

RJ highlights an original intersectional lens on integrative, rather than additive, impacts of racism, sexism, misogyny, transphobia, homophobia, and classism in reproductive politics [12]. As a praxis-based framework, RJ emphasizes application, in that “intersectionality is the process; human rights are the goal” [13]. Although there are limits to rights-based strategies [14], RJ recognizes that the legal rights are meaningless without ensuring practical access to reproductive health care. The RJ movement continues to reposition public debates and practice through activism, academia, research, and advocacy. It has since been taken up as a framework for policy development in international contexts [15, 16], with sexual and reproductive health activists in Canada calling for an adoption of a RJ framework into policymaking [17, 18].

## Reproductive violence and ART

In applying an RJ framework there needs to be awareness of critiques and cautions that RJ has made in relation to ART. This includes a history of eugenics and reproductive control using ART [9, 19, 20], a current “slippery slope” between eugenics and ART in gene-selection [21], the global commercialization of pregnancy and exploitation of poor people with wombs [10, 22, 23], and material economic access to ART [24–26]. These are important considerations when applying an RJ framework to ART and SRHR among BIPOC communities.

By engaging with critiques of ART, we can better understand constitutive elements of reproductive capacity and access. It is a call to consider historical and ongoing conditions of racial violence and reproductive control of racialized people’s bodies. Black and women of colour feminists have aptly written about reproduction and regulation of Black, Indigenous, and people of colour’s bodies as central to intersectional oppression and health inequities, concealed into the ordinary present and unacknowledged past [9, 19, 20, 27]. In Canada and the United States, ART exists within an oppressive history of reproductive technologies. The eugenics movement has been described as a political and scientific response to increase in populations that challenged white dominance, in turn, predisposing biological inferiority [28]. Subsequently, the birth control movement was legitimized through eugenics and racist population control of Black, Indigenous, and people of colour in the US and Canada [9, 19, 20]. Birth control campaigns were targeted to control the birth rates of Black, Indigenous, and people of colour [9, 19]. In 1921, Margaret Sanger, founder of the birth control movement, asserted that “Birth Control is not merely of eugenic value, but is practically identical in ideal with the final aims of Eugenics” as “the most urgent problem today is how to limit and discourage the overfertility of the mentally and physically defective” [29]. Additionally, the historical arc of gynecology and advancement of reproductive medicine is built on experimental surgeries of enslaved Black women [30]. Thus, reproductive violence and control have produced and upheld white supremacy, racial hierarchy, and ableism under guises of enhancing national strength and public health.

However, reproductive violence is not just a thing of the past in Canada. Legislated and non-legislated coerced sterilizations were and are currently used as a form of mass birth control for Black, Indigenous, and people of colour, as well as LGBTQ2SIA+ people [9, 22, 31–35]. In 2017, a class action lawsuit was launched by Indigenous women in Saskatchewan who reported experiences of coerced and forced sterilization [36]. Since then, over 100 Indigenous women across Canada have come forward between 2015 and 2019 to report their experiences [34] with more and ongoing research being conducted. In 2018, the United Nations Committee Against Torture officially recognized coerced and forced sterilization of Indigenous women in Canada as torture and called on Canada to investigate all allegations [37]. Subsequently, in 2019, Canada’s Standing Senate Committee on Human Rights studied evidence on continued forced and coerced sterilization of persons in Canada, including intersex people, trans people, women and girls with disabilities, Indigenous women, and Black women [38]. In a report released by the Senate Committee in June 2021, preliminary studies identified that Indigenous women continue to experience forced and coerced sterilization, as well as, poor people, people living with disabilities, Black women, racialized and ethnic women, and people living with HIV [39]. As a result, speakers providing Evidence for the Standing Senate Committee of Human Rights called for bringing an intersectional framework into sexual and reproductive rights that includes centering issues of racism, discrimination, lack of education, poverty, and unemployment [38]. In other words, a reproductive justice approach that utilizes intersectionality as the process and human rights as the goal, is needed.

## Access to ART: whose reproduction is centered?

Currently, the majority of ART is privatized with access being a highly stratified and stratifying process [40–42]. If ART is accessible to some groups more than others, it reinforces existing structures and forms of power and privilege where dominant groups are “empowered to nurture and reproduce, while others are disempowered” ([41] p. 3). As members of more than one marginalized group, BIPOC LGBTQ2SIA+ people experience compounded forms of oppression, including racism, homophobia, and heterosexism [43, 44]. Experiences of reproduction, reproductive capacity and decision making may be informed by multiple structures of regulation including racialization, the process of ascribing hierarchal racial identity and meaning to a social group [45–47]; heteronormativity, a system of regulation that ascribes power, privilege, and normative status to heterosexuality, sexual behaviours, and family structures [48, 49]; and cisnormativity, a system of regulation that assumes a person’s gender identity matches their biological sex [50–52]. These hierarchal power structures intersect and create compounded barriers to BIPOC LGBTQ2SIA+ people’s access to ART.

Sociotechnical innovation of reproductive access and ART research have been uneven with respect to meeting the unique reproductive needs of LGBTQ2SIA+ people. ART was initially designed to provide “infertile/low fertility” heterosexual married couples with options to conceive children [53–55]. This conception of ART continues to center infertility and heterosexual couples as the imagined user in research, treatment protocol, and clinic spaces [5, 56]. In Canada, LGBTQ2SIA+ people have been historically denied parenthood with fertility clinics refusing to provide services to couples that were not heterosexual and married [57]. Additionally, studies have shown that LGBTQ2SIA+ people experience heteronormative and cisnormative assumptions at fertility clinics regarding linkages of body parts, sexuality, and sexual practice [5, 57]. The lack of education and training on LGBTQ2SIA+ positive services for ART providers has led to barriers for LGBTQ2SIA+ people who do not fit a heteronormative model of ART [5, 56]. This is an issue when up to 25% of fertility clinic users in urban areas of Canada identify as LGBTQ2SIA + [58].

## Who are ART users in Canada?

Regulation of ART and funding for fertility are under provincial jurisdiction in Canada [59]. Currently, Ontario has the most inclusive publicly funded fertility program. The Ontario Fertility Program (OFP) is aimed at addressing accessibility and coverage of fertility services [60]. Under the OFP, individuals under the age of 43 with a uterus and valid Ontario Health Insurance Plan (OHIP) card are eligible for one funded IVF cycle regardless of sex, gender, sexual orientation, and family status [60]. Yet it is unknown who the program is targeting because there is no publicly available sociodemographic and distribution of funding data. In creating OFP, The Ministry of Health and Long-Term care did not provide any guidelines or principles of prioritization for fertility clinics to distribute resources [61]. This has resulted in non-standardized waitlists with varying mechanisms of prioritization and a two-tiered system where those who could afford to pay can be put on the waitlist and still opt for privately funded IVF [61, 62]. The majority of fertility clinics in Ontario are privatized with no overarching provincial legislation or standardization to govern the practice or fees associated [5]. Overall, the availability of data and non-standardized priority mechanisms make it difficult to assess the publicly funded program and accessibility of fertility care.

Overall, fertility clinic data in Canada is voluntarily reported and stored through the Canadian Assisted Reproductive Technologies Register [63]. As clinics are not mandated to report data, reports stemming from this database disclose that the data may not be representative or completely accurate [63, 64]. Existing reports from the database do not include patient demographics beyond age. The only inclusion that may be related to sexuality and family structure is ‘no male partner’ under ‘reasons for treatment’. However, this presumes heteronormativity, cisnormativity, and a two-person couple, while not providing any further information. The lack of reported data on race, gender, sexuality, and class obstructs research on who the users are, who is excluded, and what are barriers to access.

In contrast to the Canadian context, fertility clinics in the US performing ART are required to report data to the Centers for Disease Control and Prevention through the Society for Assisted Reproductive Technology Clinic Outcome Reporting System [65]. Using this database, studies have shown that there are racial and ethnic disparities in ART access and treatment outcomes [26, 66, 67]. These disparities have also been shown to be increasing [68]. Even as less than 65% of IVF cycles report on race/ethnicity, studies suggest significant racial/ethnic disparities in IVF outcomes [67]. Lastly, collected data on education level and income have also identified the cost of ART as barriers to access and treatment [66].

## Research in ART: whose reproduction has been centered?

Present research on reproductive technologies and fertility clinics predominantly centers on white, cisgender, heterosexual couples with socioeconomic privilege. In addition, the rare research study exploring LGBTQ2SIA+ participants’ experiences with reproductive access have included primarily white, cisgender lesbian and bisexual women with relatively high levels of education and income [5, 69, 70]. Limited available data in Canada and the United States that includes broader patient populations suggests access to assisted reproductive technologies is uneven across intersecting social categories of race, gender, sexuality, and class [4, 6]. A frequently mentioned limitation is centering of whiteness in research development and participant recruitment [71, 72]. In turn, this upholds structural racism and white supremacy when whiteness and white experiences are the norm, while racial inequities exist because of differential access to resources and opportunities that constitute health [73].

## Implementing RJ in ART access and research

An RJ approach to ART includes understanding and dismantling structures that seek to control the reproductive capacities of marginalized communities, while empowering accessibility and upholding white supremacy and heteronormativity. Implementing RJ in ART access and research would include asking questions like who gets access to ART? What are present and historical conditions of ART that impact access? What socio-political factors impact reproductive access? Is the right to parent children in safe and healthy environments supported [10]? Additionally, applying an RJ lens in ART, and SRHR broadly, would include actively putting an end to coerced and forced sterilization; holding systems and institutions accountable to reproductive violence in the past and present; recognizing barriers that still impact access and potentially further distrust; working on building trust; and recognizing that social locations and relations to community matter. RJ includes recognizing that the ART user is more than who is seen at the fertility clinic.

Beyond data collection on socio-demographics, the lived realities of reproductive capacity and decision making among BIPOC LGBTQ2SIA+ communities should be considered in research, clinical work, and policy development. Experiences of integrative social locations including race, class, gender, sexuality, family structure, and access to health care [13] are constituted by historically and ongoing inequitable practices, reproductive violence, and regulation of bodies, sexuality, and population. The barriers to healthcare and fertility services are beyond the immediate encounters, as they are informed by lived realities that constitute reproductive capacity, decision making, conditions before and after birthing (i.e., access to resources, access to healthcare, familial elements, cultural values, educational opportunities).

## Conclusion

As a family formation process, ART brings interconnections of sexuality, gender, race, socioeconomic status, family structure, and access to healthcare into play. Applying RJ as an approach to ART for BIPOC LGBTQ2SIA+ communities and other users necessitates attention towards complex constitutive elements that impact reproductive capacities and decisions that are not often at the forefront of reproductive health discussions. As a foundational framework, reproductive justice can advance our understandings and attempts to reduce sexual and reproductive health inequities among BIPOC (and) LGBTQ2SIA + communities in Canada.

## Data Availability

Not applicable.

## Abbreviations

ART: Assisted reproductive technology
BIPOC: Black, Indigenous, and people of colour
LGBTQ2SIA+: Lesbian, gay, bisexual, transgender, queer, two-spirit, intersex, asexual, and (+) sexual and gender diverse people
OHIP: Ontario Health Insurance Plan
RJ: Reproductive justice

## References

[1] Canadian Fertility and Andrology Society. Assisted Reproductive Technologies (ART) in Canada: 2015 results from the Canadian ART Register (CARTR) 2016. https://cfas.ca/canadian-art-register.html. Accessed 2 Jun 2021.

[2] Bushnik T, Cook JL, Yuzpe AA, Tough S, Collins J (2012). Estimating the prevalence of infertility in Canada. Hum Reprod.

[3] Mamo L (2013). Queering the fertility clinic. J Med Hum.

[4] Mamo L, Alston-Stepnitz E (2015). Queer intimacies and structural inequalities: new directions in stratified reproduction. J Fam Issues.

[5] Ross LE, Tarasoff LA, Anderson S, Epstein R, Marvel S, Steele LS (2014). Sexual and gender minority peoples’ recommendations for assisted human reproduction services. J Obstet Gynaecol Can.

[6] Marvel S. Polymorphous reproductivity and the critique of futurity: toward a queer legal analytic for fertility law. Jindal Glob Law Rev. 2013;4(2):296–314.

[7] Combahee River Collective. A Black Feminist Statement. 1977.

[8] Crenshaw K (1990). Mapping the margins: intersectionality, identity politics, and violence against women of color. Stan L Rev.

[9] Racism DA, Control B, Rights R, Davis A (1982). Women, race and class.

[10] Ross L, Solinger R (2017). Reproductive justice: an introduction.

[11] Asian Communities for Reproductive Justice. A new vision for advancing our movement for reproductive health, reproductive rights and reproductive justice. Oakland, CA; 2005.

[12] Ross L (2006). Understanding reproductive justice: transforming the pro-choice movement. Off Our Backs.

[13] Ross LJ (2017). Reproductive justice as intersectional feminist activism. Souls.

[14] Rebouché R (2017). Reproducing rights: the intersection of reproductive justice and human rights. UC Irvine L Rev.

[15] Macleod CI, Beynon-Jones S, Toerien M (2017). Articulating reproductive justice through reparative justice: case studies of abortion in Great Britain and South Africa. Cult Health Sex.

[16] Chiweshe M, Mavuso J, Macleod C (2017). Reproductive justice in context: South African and Zimbabwean women’s narratives of their abortion decision. Fem Psychol.

[17] Kirby J. A broad vision for reproductive justice. Action Canada for sexual health and rights. 2017. https://www.actioncanadashr.org/news/2017-12-22-broad-vision-reproductive-justice. Accessed 2 Jun 2021.

[18] Violence on the land, violence on our bodies: Building an Indigenous response to environmental violence [report on the Internet]. California: Women’s Earth Alliance & Native Youth Sexual Health Network; 2016. http://landbodydefense.org/uploads/files/VLVBReportToolkit2016.pdf?. Accessed 2 Jun 2021.

[19] Murphy M (2012). Seizing the means of reproduction: entanglements of feminism, health, and technoscience.

[20] Roberts DE (1997). Killing the black body: race, reproduction, and the meaning of liberty.

[21] Roberts DE (2009). Race, gender, and genetic technologies: a new reproductive dystopia?. Signs J Women Cult Soc..

[22] Weinbaum AE (2019). The afterlife of reproductive slavery: biocapitalism and black feminism’s philosophy of history.

[23] Vora K (2012). Limits of “labor”: Accounting for affect and the biological in transnational surrogacy and service work. S Atl Q.

[24] Chandra A, Martinez GM, Mosher WD, Abma JC, Jones J. Fertility, family planning, and reproductive health of US women; data from the 2002 National Survey of Family Growth. 2005. 16532609

[25] Chambers GM, Sullivan EA, Ishihara O, Chapman MG, Adamson GD (2009). The economic impact of assisted reproductive technology: a review of selected developed countries. Fertil Steril.

[26] Fujimoto VY, Luke B, Brown MB, Jain T, Armstrong A, Grainger DA, Hornstein MD, Society for Assisted Reproductive Technology Writing Group (2010). Racial and ethnic disparities in assisted reproductive technology outcomes in the United States. Fertil Steril.

[27] Gumbs AP, China M, Williams M (2016). Revolutionary mothering: love on the front lines.

[28] Somerville S (1994). Scientific racism and the emergence of the homosexual body. J Hist Sex.

[29] Sanger M (1921). The eugenic value of birth control propaganda. Birth Control Rev.

[30] Owens DC (2017). Medical bondage: race, gender, and the origins of American gynecology.

[31] Lowik A (2018). Reproducing eugenics, reproducing while trans: the state sterilization of trans people. J GLBT Fam Stud.

[32] Kline W (2001). Building a better race: gender, sexuality, and eugenics from the turn of the century to the baby boom.

[33] Rich A (1980). Compulsory heterosexuality and lesbian existence. Signs.

[34] Shawana C, Ryan C, Ali A (2021). Forced or coerced sterilization in Canada: an overview of recommendations for moving forward. Int J Indig Health..

[35] Stote K (2012). The coercive sterilization of aboriginal women in Canada. Am Indian Cult Res J.

[36] M.R.L.P and S.A.T. v The Attorney General of Canada, the Government of Saskatchewan, Saskatchewan Health Authority, Athabasca Health Authority, Dr. Kristine Mytopher, Dr. Ahmed Ezzat, Dr. Ian Lund, John Doe, and Jane Doe, QB 1485 (2017), filed 16 February 2018 (https://cbaapps.org/ClassAction/PDF.aspx?id=10097).

[37] The Committee against torture. Concluding observations on the seventh periodic report of Canada. Geneva, Switzerland: United Nations Committee Against Torture; 2018 Dec 21. http://docstore.ohchr.org/SelfServices/FilesHandler.ashx?enc=6QkG1d%2FPPRiCAqhKb7yhsglSZMQd1BoEakgym8DLljp%2FtVZwAcP32UhceoEv6s9EFDnHa%2FfIXxFR9KNVY4qkr3X7%2FaP5eVqCmw6nDLJyD3dA5iGzIWJ0XfsLEbi0yIvz.

[38] Canada, Parliament, House of Commons, Standing Committee on Human Rights, Evidence of Proceedings, 42nd Parl, 1st Sess, No 42 (15 May 2019).

[39] Standing Senate Committee on Human Rights. Forced and coerced sterilizations of persons in Canada. Ottawa, ON: Senate of Canada; 2021.

[40] Barnes L, Fledderjohann J (2020). Reproductive justice for the invisible infertile: a critical examination of reproductive surveillance and stratification. Sociol Compass.

[41] Ginsburg FD, Rapp R, Reiter RR, Rapp RR (1995). Conceiving the new world order: the global politics of reproduction.

[42] Colen S (1986). Stratified reproduction: the case of domestic workers in America.

[43] Pollitt AM, Reczek C, Umberson D. LGBTQ-parent families and health. In: LGBTQ-parent families. Cham: Springer; 2020. p. 125–40.

[44] Warner M (2002). Publics and counterpublics. Publ Cult.

[45] Delgado R, Stefancic J (2017). Critical race theory: an introduction.

[46] Omi M, Winant H (2018). Racial formation in the United States.

[47] Harris CI (1993). Whiteness as property. Harvard Law Rev.

[48] Somerville SB (2020). The Cambridge companion to queer studies.

[49] Cohen CJ (1997). Punks, bulldaggers, and welfare queens: the radical potential of queer politics?. GLQ J Lesbian Gay Stud..

[50] Snorton CR (2017). Black on both sides: a racial history of trans identity.

[51] Preciado B (2013). Testo junkie: sex, drugs, and biopolitics in the pharmacopornographic era.

[52] Serano J. Trans-misogyny primer. Whipping Girl. 2012;3.

[53] Garwood E (2016). Reproducing the homonormative family: neoliberalism, queer theory and same-sex reproductive law. J Int Women's Stud.

[54] Nordqvist P (2008). Feminist heterosexual imaginaries of reproduction: lesbian conception in feminist studies of reproductive technologies. Fem Theory.

[55] Franklin S (2002). Embodied progress: a cultural account of assisted conception.

[56] Hayman B, Wilkes L, Halcomb E, Jackson D (2013). Marginalised mothers: lesbian women negotiating heteronormative healthcare services. Contemp Nurse.

[57] Epstein R (2018). Space invaders: queer and trans bodies in fertility clinics. Sexualities.

[58] Green D, Tarasoff LA, Epstein R. Meeting the assisted human reproduction (AHR) needs of lesbian, gay, bisexual, trans and queer (LGBTQ) people in Canada: a fact sheet for AHR service providers. Toronto, ON: LGBTQ Parenting Network. 2012.

[59] Guichon J, Mitchell I, Doig C (2013). Assisted human reproduction in common law Canada after the Supreme Court of Canada reference: moving beyond regulation by colleges of physicians and surgeons. Can J Women Law.

[60] Government of Ontario. Get fertility treatments. Ontario, Canada: Government of Ontario; 2017. https://www.ontario.ca/page/get-fertility-treatments. Accessed 7 Jul 2021.

[61] Assal A, Jones CA, Gotz T, Shah BR (2019). The impact of the Ontario fertility program on duplicate fertility consultations. Healthc Policy.

[62] Gotz T, Jones C (2017). Prioritization of patients for publicly funded IVF in Ontario: a survey of fertility centres. J Obstet Gynaecol Can.

[63] Canadian Assisted Reproductive Technologies Registry (CARTR) Plus. Preliminary treatment cycle data for 2019. Ottawa, ON: Better Outcomes Registry & Network Ontario; 2021 February.

[64] Canadian Assisted Reproductive Technologies Registry (CARTR) Plus. Final treatment cycle and pregnancy outcome data for 2018. Ottawa, ON: Better Outcomes Registry & Network Ontario; 2021 February.

[65] Sunderam S, Kissin DM, Zhang Y, Jewett A, Boulet SL, Warner L, Kroelinger CD, Barfield WD (2020). Assisted reproductive technology surveillance—United States, 2017. MMWR Surveill Summ.

[66] Quinn M, Fujimoto V (2016). Racial and ethnic disparities in assisted reproductive technology access and outcomes. Fertil Steril.

[67] Wellons MF, Fujimoto VY, Baker VL, Barrington DS, Broomfield D, Catherino WH, Richard-Davis G, Ryan M, Thornton K, Armstrong AY (2012). Race matters: a systematic review of racial/ethnic disparity in Society for Assisted Reproductive Technology reported outcomes. Fertil Steril.

[68] Chandra A, Copen CE, Stephen EH. Infertility service use in the United States: data from the National Survey of Family Growth, 1982–2010: US Department of Health and Human Services, Centers for Disease Control and Prevention; 2014.

[69] Marvel S, Tarasoff LA, Epstein R, Green D, Steele LS, Ross LE, Milne C, Lee IB, Lemmens TF, Martin A (2017). Listening to LGBTQ people on assisted human reproduction: access to reproductive material, services, and facilities. Regulating creation.

[70] Mamo L (2007). Queering reproduction: achieving pregnancy in the age of technoscience.

[71] Karpman HE, Ruppel EH, Torres M (2018). “It wasn't feasible for us”: queer women of color navigating family formation. Fam Relat.

[72] Ahmed S (2007). A phenomenology of whiteness. Fem Theory.

[73] El-Mowafi IM, Yalahow A, Idriss-Wheeler D, Yaya S (2021). The politest form of racism: sexual and reproductive health and rights paradigm in Canada. Reprod Health.

